# Macrophage Migration Inhibitory Factor Acts as the Potential Target of a Newly Synthesized Compound, 1-(9′-methyl-3′-carbazole)-3, 4-dihydro-β-carboline

**DOI:** 10.1038/s41598-019-38590-y

**Published:** 2019-02-14

**Authors:** Pin-Hao Ko, Ya-Ching Shen, Kaliyappan Murugan, Chiung-Wei Huang, Govindan Sivakumar, Pinki Pal, Chia-Ching Liao, Kai-Shin Luo, Eric Y. Chuang, Mong-Hsun Tsai, Liang-Chuan Lai

**Affiliations:** 1 Graduate Institute of Physiology, College of Medicine, National Taiwan University, Taipei, Taiwan; 2 School of Pharmacy, College of Medicine, National Taiwan University, Taipei, Taiwan; 3 Department of Physiology, College of Medicine, Kaohsiung Medical University, Kaohsiung, Taiwan; 4 Department of Ophthalmology, Taipei Tzu Chi Hospital, Buddhist Tzu Chi Medical Foundation, Taipei, Taiwan; 5 Graduate Institute of Biomedical Electronics and Bioinformatics, National Taiwan University, Taipei, Taiwan; 6 Bioinformatics and Biostatistics Core, Center of Genomic and Precision Medicine, National Taiwan University, Taipei, Taiwan; 7 Institute of Biotechnology, National Taiwan University, Taipei, Taiwan

## Abstract

For a newly synthesized compound, identifying its target protein is a slow but pivotal step toward understand its pharmacologic mechanism. In this study, we systemically synthesized novel manzamine derivatives and chose 1-(9**′**-methyl-3**′**-carbazole)-3, 4-dihydro-β-carboline (MCDC) as an example to identify its target protein and function. MCDC had potent toxicity against several cancer cells. To identify its target protein, we first used a docking screen to predict macrophage migration inhibitory factor (MIF) as the potential target. Biochemical experiments, including mutation analysis and hydrogen-deuterium exchange assays, validated the binding of MCDC to MIF. Furthermore, MCDC was shown by microarrays to interfere with the cell cycle of breast cancer MCF7 cells. The activated signaling pathways included AKT phosphorylation and S phase-related proteins. Our results showed MIF as a potential direct target of a newly synthesized manzamine derivative, MCDC, and its pharmacologic mechanisms.

## Introduction

The natural world is a major source of small molecules for development as novel pharmaceuticals. Previously, we isolated several small molecules, manzamines, from a Formosan marine sponge, *Haliclona* sp.^1^. Manzamines are members of the β-carboline alkaloids, isolated from sponges and other marine microorganisms^2,3^. To examine the pharmacologic mechanisms of these novel compounds, one of the crucial steps is to identify their target protein(s). Since a small molecule usually has potential high-affinity protein binding partners, it often takes tremendous effort and time to search for the direct target protein of a new compound. For example, the adenanthin-biotin-streptavidin system was used to identify adenanthin’s direct target proteins, peroxiredoxin I and II^4^. In this case, the process was slow, and it was difficult to get a comprehensive view of the function of adenanthin in cells.

There are two main approaches to identify the pairing between small molecules and their target proteins. One is to screen thousands of compounds in an established library against a known target protein^5^. The other is to synthesize a new compound and screen cellular binding protein(s) against the small molecule of interest^6,7^. In this study, we took the latter approach to search for the target protein(s) of newly synthesized manzamine-derived compounds. Specifically, we used bioinformatics approaches to expedite the process of understanding the pharmacologic mechanisms of the manzamine derivatives in a systematic way.

Previous data showed that manzamine A-derived compounds, such as 1-substituted carbazolyl-1, 2, 3, 4-tetrahydro-β-carboline and carbazolyl-3, 4-dihydro-β-carboline, showed significant anticancer activities against colon adenocarcinoma DLD cells, lung large cell carcinoma NCI-H661 cells, and hepatoma HepG_2_/A_2_ cells^8^. Also, elongation of the alkyl chain resulted in a decrease in these activities, although the relationship between the number of carbons in the side chain on the N atom in the carbazole and the anticancer activity of the derivatives remained unclear^8^. In addition, although some studies showed that manzamine derivatives had many potential pharmacologic functions^9–13^, their target proteins and underlying binding mechanism still remained elusive.

Therefore, to understand the structure-activity relationship of manzamines in this study, we synthesized more derivatives with a modified chemical structure at the relevant position on β-carboline and examined their cytotoxicity. We chose the manzamine-derived compound 1-(9′-methyl-3′-carbazole)-3, 4-dihydro-β-carboline (MCDC) as an example to demonstrate the possibility of rapid identification of the target protein(s) of a newly synthesized compound.

To identify the target protein(s) for our new derivative, we took advantage of virtual screening to search all potential targets simultaneously by calculating the free energy of docking between proteins in the protein data bank (PDB) and manzamine derivatives^14,15^. This method identified macrophage migration inhibitory factor (MIF) as the lowest energy binding partner of MCDC. The proposed MIF-MCDC interaction was assessed via docking simulations, hydrogen-deuterium exchange experiments, and mutation of the simulated binding site. To understand the effects of manzamine derivatives on cells at the genomic level, transcriptome profiling was examined by microarrays, and the function of differentially expressed genes was analyzed by pathway analysis. Although the direct target of MCDC may not be only one, this method rapidly identified MIF as one of the potential direct targets of MCDC and elucidated its primary pharmacologic mechanisms.

## Results

### Synthesis of 1-substituted carbazolyl-1, 2, 3, 4-tetrahydro-β-carboline and carbazolyl-3, 4-dihydro-β-carboline derivatives

**Figure 1:**
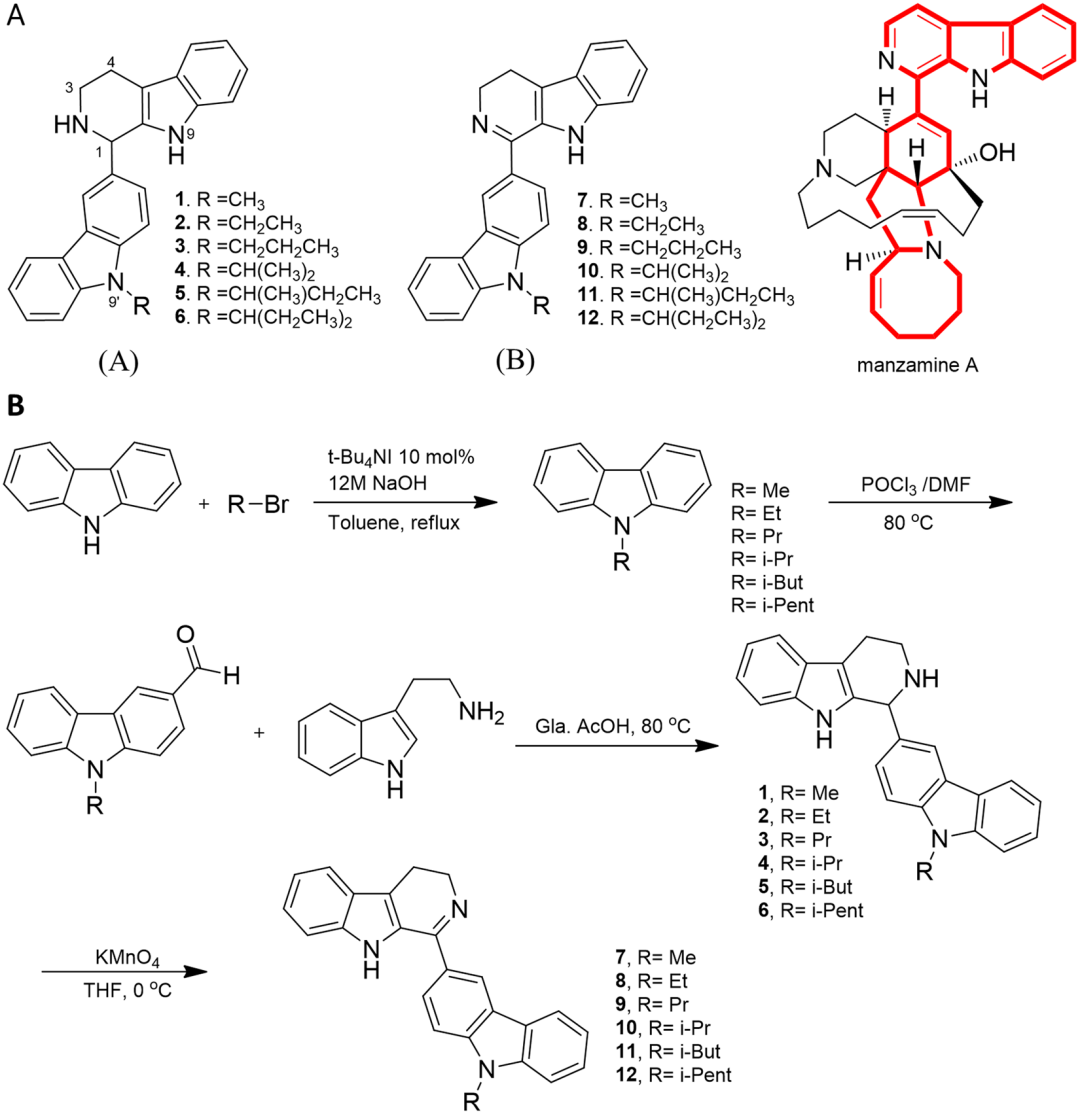
Synthesis of 1-substituted carbazolyl-1, 2, 3, 4-tetrahydro-β-carboline and carbazolyl-3, 4-dihydro-β-carboline derivatives. (**A**) Chemical structures of all 12 β-carboline derivatives. **1**: 1-(9′-methyl-3′-carbazole)-1, 2, 3, 4-tetrahydro-β-carboline; **2**: 1-(9′-ethyl-3′-carbazole)-1, 2, 3, 4-tetrahydro-β-carboline; **3**: 1-(9′-propyl-3′-carbazole)-1, 2, 3, 4-tetrahydro-β-carboline; **4**: 1-(9′-isopropyl-3′-carbazole)-1, 2, 3, 4-tetrahydro-β-carboline; **5**: 1-(9′-[1″-methyl]propyl-3′-carbazolyl)-1, 2, 3, 4-tetrahydro-β-carboline; **6**: 1-(9′-[1″-ethyl]propyl-3′-carbazole)-1, 2, 3, 4-tetrahydro-β-carboline; **7**: 1-(9′-methyl-3′-carbazole)-3, 4-dihydro-β-carboline; **8**: 1-(9′-ethyl-3′-carbazole)-3, 4-dihydro-β-carboline; **9**: 1-(9′-propyl-3′-carbazole)-3, 4-dihydro-β-carboline; **10**: 1-(9′-isopropyl-3′-carbazole)-3, 4-dihydro-β-carboline; **11**: 1-(9′[1″-methyl]propyl-3′-carbazole)-3, 4-dihydro-β-carboline; **12**: 1-(9′-[1″-ethyl]propyl-3′-carbazole)-3, 4-dihydro-β-carboline. The core structure of manzamine A is highlighted in red. (**B**) Preparation scheme of compounds **1–12**.

Previous studies showed that elongation of the alkyl chain of 1-substituted carbazolyl-1, 2, 3, 4-tetrahydro-β-carboline and carbazolyl-3, 4-dihydro-β-carboline, both manzamine A-derived compounds, resulted in decreased anticancer activity^8^. Thus, with the aim of studying the structure-activity relationship to understand and optimize the biological activity of these compounds, we attempted to derivatize the essential part of manzamine A (red color in Fig. 1A) and vary the length of the *N*-alkyl side chain on the carbazole ring. Therefore, we synthesized six additional new compounds (1, 4, 6, 7, 10, and 12 in Fig. 1A). In total, we synthesized six derivatives of both 1-substituted carbazolyl-1, 2, 3, 4-tetrahydro-β-carboline (group A) and carbazolyl-3, 4-dihydro-β-carboline (group B). The process of synthesis is depicted in Fig. 1B. Briefly, *N*-alkylcarbazoles were prepared by *N*-alkylation of the carbazole with an alkyl halide. A Vilsmeier-Haak reaction^16^ followed by Pictet-Spengler cyclization^17,18^ yielded compounds 1–6, and oxidation^19^ furnished the desired compounds 7–12, respectively.

### 1-(9′-methyl-3′-carbazole)-3, 4-dihydro-β-carboline (MCDC) reduced viability in four cancer cell lines

Previous data showed that some manzamine derivatives had cytotoxic effects against human cancer cells^1^. To examine the cytotoxic effects of our 12 newly synthesized derivatives, we performed Calcein AM viability assays on four cancer cell lines from lung (A549, H1299), liver (HepG2), and breast (MCF7) with the dose range of each compound from 0, 1, 10 and 100 μM (Supplementary Fig. S1). Table 1 summarizes the viability information at the dose of 10 μM. Treatment with compounds 2, 3, 4, and 7 reduced the percentage of viable cells to <25% in all tested cell lines (Table 1, bold font). Also, compound **7** was the most effective drug in group B. Other members in group B (compound 8–12 in Table 1), with longer *N*-alkyl side chain than compound **7**, showed less cytotoxic effect (Table 1). In this study, the newly synthesized compound 7, 1-(9′-methyl-3′-carbazole)-3, 4-dihydro-β-carboline (MCDC) in group B, was taken as an example to demonstrate our methodology for rapid investigation of the structure-activity relationship.

**Table 1:** Cytotoxicity of 12 β-carboline derivatives (10 μM) in four cancer cell lines.

Compound No.	A549	H1299	HepG2	MCF7
1	54.9 ± 14.1^†^	77.9 ± 26.3	32.5 ± 8.8	43.4 ± 16.0
2	**15.8** ± **8.1**	**12.8** ± **2.1**	**21.9** ± **1.6**	**16.4** ± **4.2**
3	**14.8** ± **9.7**	**15.0** ± **6.2**	**21.1** ± **2.1**	**14.9** ± **1.2**
4	**16.8** ± **7.4**	**12.5** ± **3.6**	**21.0** ± **2.9**	**20.3** ± **5.3**
5	33.4 ± 7.4	41.2 ± 20.2	21.9 ± 2.6	29.1 ± 10.0
6	28.2 ± 4.0	17.1 ± 8.4	18.3 ± 1.5	28.0 ± 5.5
7	**16.6** ± **7.7**	**15.3** ± **10.3**	**22.9** ± **3.0**	**20.9** ± **8.5**
8	44.6 ± 7.0	51.0 ± 21.6	21.6 ± 7.2	39.9 ± 8.5
9	51.0 ± 5.3	59.8 ± 13.2	27.3 ± 5.4	60.7 ± 5.3
10	67.4 ± 21.6	71.3 ± 17.0	28.8 ± 14.2	52.3 ± 13.7
11	58.1 ± 25.2	45.1 ± 3.0	27.5 ± 9.9	65.2 ± 7.6
12	52.7 ± 4.6	54.6 ± 3.1	35.0 ± 1.7	64.9 ± 3.2

^†^Cell viability (%) was measured using Calcein AM assays. All data are presented as the average percentage of viable cells ± SD.

In order to calculate the IC_50_ value of MCDC accurately, the viability of A549 and MCF7 cells treated with a narrower range of MCDC concentration (0–8 μM) was measured using MTT assays. The IC_50_ values of MCDC in A549 (Supplementary Fig. S2A) and MCF7 (Supplementary Fig. S2B) cells were about 5.04 ± 0.29 and 5.48 ± 0.13 μM, respectively, 48 h after treatment. Complete physical, spectroscopic, and spectrometric data for MCDC are listed in Table 2.

**Table 2:** Physical, spectroscopic, and spectrometric data of 1-(9′-methyl-3′-carbazole)-3, 4-dihydro-β-carboline (MCDC).

Characterization	Data summary
Physical form	Yellow solid
Melting temperature	187 °C
UV λ_max_	236, 287 nm
IR (KBr) ν_max_	3050, 2923, 1593, 1491, 1368, 1272, 744 cm^−1^
^1^H NMR (CDCl_3_)	*δ*_H_ 4.00 (2 H, t, *J* = 7.8 Hz, H-3), 2.98 (2 H, t, *J* = 7.8 Hz, H-4), 7.68 (1 H, d, *J* = 7.6 Hz, H-5), 7.20 (1 H, m, overlap, H-6), 7.25 (1 H, m, overlap, H-7), 7.40 (1 H, m, overlap, H-8), 7.38 (1 H, m, overlap, H-1′), 7.88 (1 H, dd, *J* = 8.4, 1.4 Hz, H-2′), 8.47 (1 H, s, H-4′), 7.29 (1 H, m, overlap, H-5′), 7.50 (1 H, d, *J* = 7.0 Hz, H-6′), 7.23 (1 H, m, overlap, H-7′), 8.05 (1 H, d, *J* = 7.6 Hz, H-8′), 3.81 (3 H, s, CH_3_)
^13^C NMR (CDCl_3_)	*δ*_C_ 159.9 (s, C-1), 48.4 (t, C-3), 19.3 (t, C-4), 128.2 (s, C-4b), 120.0 (d, C-5), 120.6 (d, C-6), 126.1 (d, C-7), 112.1 (d, C-8), 136.5 (s, C-8a), 117.9 (s, C-9a), 108.7 (d, C-1′), 125.8 (d, C-2′), 141.9 (s, C-3′), 120.3 (d, C-4′), 122.8 (s, C-4′a), 122.7 (s, C-4′b), 108.5 (d, C-5′), 124.4 (d, C-6′), 119.3 (d, C-7′), 120.2 (d, C-8′), 125.7 (s, C-8′a), 141.4 (s, C-9′a), 29.1 (q, CH_3_)
ESIMS *m*/*z*	350 (100, M + H^+^)
HREIMS *m*/*z*	350.1657 ([M + H]^+^, calcd. for C_24_H_20_N_3_, 350.1652)

1D NMR spectra were recorded by Bruker AV-400 spectrometer using CDCl_3_ as internal standard. Chemical shifts (*δ*) were expressed in ppm relative to CDCl_3_ signals. HRESIMS was performed on a FINNIGAN MAT 95S Mass Spectrophotometer. Hitachi U-2001 spectrophotometer was for UV spectra and Thermo Nicolet iS5 FT-IR Spectrometer was for IR spectra by using KBr pellets. Melting point was obtained by BÜCHI Melting Point B-540 *melting point* apparatus.

### MIF was a potential target protein of MCDC

Since small molecules often have many target proteins, we employed several computational methods to speed up the process of identifying potential binding partners for MCDC. We first used a web server, idTarget^20^, to screen the docking free energy of MCDC with all proteins in Protein Data Bank (PDB). However, many published protein structures in PDB did not belong to *Homo sapiens*. The results of idTarget predicted 59 proteins as MCDC target with free energy (ΔG) values less than −10.5 kcal/mol. Only 15 of them are *Homo sapiens’* proteins (Table 3). Among these predicted target proteins, macrophage migration inhibitory factor (MIF; PDB No. 3l5 s) had the lowest free energy when docked with MCDC, indicating stable binding between MIF and MCDC. Therefore, we chose MIF for the following experiments.

**Table 3:** Top 15 predicted target proteins of MCDC, obtained by docking in idTarget using the Protein Data Bank (PDB).

idTarget No.^#^	PDB No.	Human protein	ΔG^†^ (kcal/mol)
1	3l5 s	Macrophage migration inhibitory factor	−11.16
2	1xur	Matrix metalloproteinase-13	−11.03
3	3f16	Catalytic domain of human MMP12	−10.91
4	3bcj	Aldose Reductase complexed with 2S4R	−10.85
5	1gkc	MMP9-inhibitor	−10.8
6	1zq9	Dimethyladenosine transferase	−10.77
7	1xuc	Matrix metalloproteinase-13	−10.74
8	2reo	Human sulfotransferase 1C3	−10.73
9	3ipq	LXR-alpha	−10.72
10	3f17	Catalytic domain of human MMP12	−10.67
11	1pq6	LXR beta hormone receptor	−10.58
12	1i5r	Type 1 17-beta hydroxysteroid dehydrogenase	−10.57
13	3hy7	Catalytic Domain of ADAMTS-5	−10.55
14	1s9d	ARF1[delta 1–17]-GDP-MG	−10.54
15	1hs6	Leukotriene A4 hydrolase	−10.51

^#^idTarget No. is listed in ascending order of the free energy of docking with MCDC.

^†^ΔG is the free-energy difference (ΔG) between MCDC and candidate protein.

**Figure 2:**
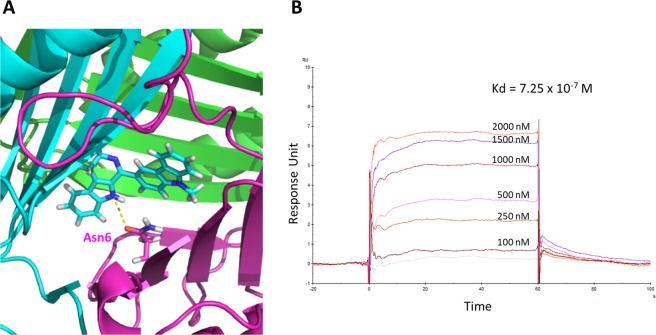
Binding analysis between MCDC and recombinant human MIF protein. (**A**) The predicted interaction between MIF and MCDC. MIF (PDB ID: 3L5S) is presented as a homotrimer (labeled in blue, green, and pink and rendered as ribbons). Binding of MCDC (stick rendering) to Asn6 of one MIF subunit (the pink one) via a predicted hydrogen bond (the yellow dashed line) is shown. The structure was computed by Discovery Studio 3.0. (**B**) Representative biosensorgram between MCDC and MIF. Affinity binding of MCDC to MIF was measured by real-time surface plasmon resonance (Biacore analysis).

Next, to identify the amino acids of MIF involved in the interaction with MCDC, Discovery Studio 3.0 was used to predict the docked site. Asn6 of MIF was predicted to be the docking site (Fig. 2A). A hydrogen bond (dashed yellow line in Fig. 2A) formed between the asparagine and one of the nitrogen atoms in MCDC, and the distance between them was about 3.5 Å.

The kinetic binding activities between recombinant human MIF protein and MCDC (compound 7), compound 6, and compound 11 were measured by surface plasmon resonance technique. Optical biosensor surfaces were prepared by Biacore analysis system. As shown in Fig. 2B, the equilibrium dissociation constant (Kd) showed fine binding activity between MCDC and MIF (7.25 × 10^−7^ M), but the Kds of compound 6 and 11 with MIF could not be measured because the response fluctuated in a random manner as the dose of compound 6 or 11 increased (data not shown), indicating compound 6 and compound 11 were not the ligands for MIF.

**Figure 3:**
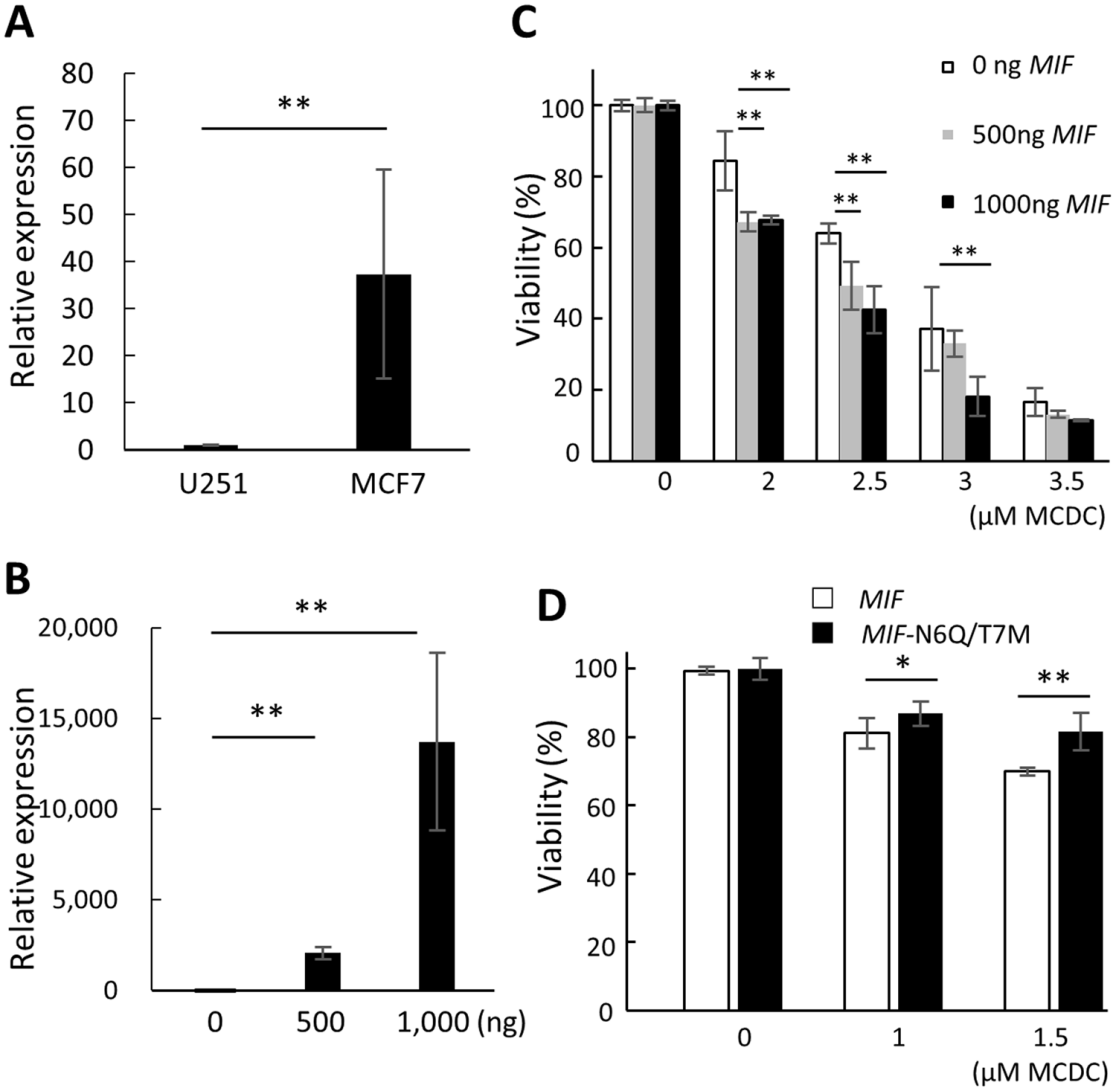
MIF enhanced the cytotoxic effects of MCDC in U251 cells. (**A**) Relative expression levels of endogenous *MIF* in MCF7 and U251 cells. The expression level of MIF was measured by qRT-PCR. Internal control: *ACTB*. The relative expression level of MCF7 cells was normalized to that of U251 cells. (**B**) Overexpression of *MIF* in U251 cells. *MIF* was overexpressed and measured by qRT-PCR 24 h after transfection with the amounts of plasmid shown. (**C**) Effects of *MIF* on drug sensitivity of MCDC in U251 cells. Different amounts of *MIF* were overexpressed in U251 cells treated with different doses of MCDC. The viability of cells was measured by MTT assays. (**D**) Effects of MIF N6Q/T7M mutant on viability of U251 cells treated with MCDC. The viability of U251 cells overexpressing MIF or MIF mutants under different doses of MCDC was measured by MTT assays. All data are presented as means ± SDs of three independent experiments. *P < 0.05; **P < 0.01 by Student’s t-test.

To validate these simulation results, we investigated the importance of Asn6 in MIF by mutating it to alanine (A) alone and by mutating amino acids to similar acidity and polarity but larger molecular size, i.e., Asn6 to glutamine (Q) and Thr7 to methionine (M), and observed the changes in cellular function. The MIF mutant was overexpressed in the human glioblastoma cell line U251, which was identified as having low endogenous *MIF* expression in the online analytic platform Cellexpress^21^ (http://cellexpress.cgm.ntu.edu.tw/), developed by our previous work. qRT-PCR was first used to confirm the endogenous *MIF* expression. As expected, the endogenous expression of *MIF* in MCF7 cells was almost forty times higher than in U251 cells (Fig. 3A). Next, various amounts of *MIF* plasmid were transfected into U251 cells (Fig. 3B) to optimize the transfection conditions.

To examine whether the expression levels of MIF were correlated with the drug sensitivity of MCDC, we overexpressed different doses of MIF to U251 cells. As shown in Fig. 3C, at the treatment of same MCDC concentration, higher amount of MIF significantly decreased cell viability using MTT assays, indicating MIF increased the drug sensitivity of MCDC.

We then transfected MIF and its mutant into U251 cells. In the presence of MCDC, U251 cells transfected with MIF-N6Q/T7M mutant, not MIF-N6A mutant (Supplementary Fig. S3), had higher viability as compared to cells expressing wild-type MIF (Fig. 3D), indicating that mutation of N6Q and T7M in MIF negatively affected the docking of MCDC and resulted in higher cell viability.

**Figure 4:**
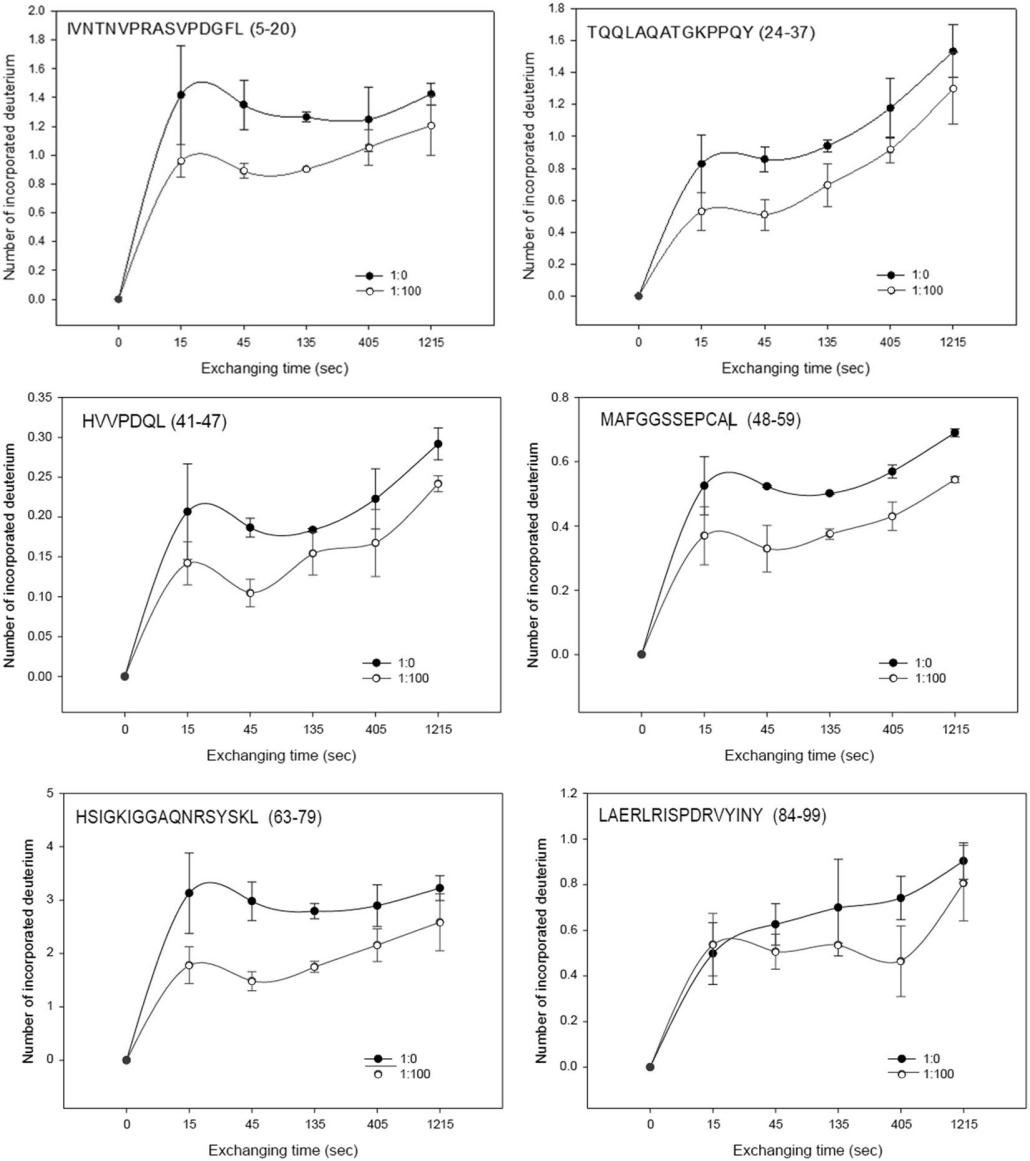
Hydrogen-deuterium exchange (HDX) assays of MIF in the presence and absence of MCDC. The deuterium uptake curves for six representative peptides are shown. Numbers in parentheses are the amino acid positions of each peptide. HDX profiles of MIF protein (1.5 pmol) in the absence (black dot) and presence (white dot) of MCDC (150 pmol) show how many deuteriums were incorporated. The y-axis is the amount of deuterium that could be incorporated to each peptide. A native detergent (0.13% of MMPC, 1-myristoyl-sn-glycero-3-phosphocholine) was used to dissolve the MCDC. The molar ratio of MIF:MCDC equals 1:100 (white dot) or 1:0 (black dot) in 0.13% of MMPC.

To further validate the simulation results, hydrogen-deuterium exchange (HDX) assays were used to examine the location of MIF’s interaction with MCDC *in vitro*. The compound MCDC is extremely hydrophobic but the HDX takes place in aqueous phase. In order to dissolve MCDC in aqueous solution for HDX, a native detergent (1-myristoyl-sn-glycero-3-phosphocholine [MMPC]) was used to dissolve MCDC without interfering with pepsin digestion and LC-MS analysis. At a molar ratio of 1:100 (MIF: MCDC = 1.5 pmol: 150 pmol in 0.13% of MMPC), MCDC blocked the exchange of hydrogen for deuterium in MIF. The protection against exchange was observed in amino acids 1–79 of MIF (Fig. 4). To ensure the protection effect of MCDC was not an artifact of MMPC, we also used DMSO (15%) to dissolve the MCDC. The results of HDX assays using DMSO as a solvent in the absence of MCDC had similar effects to those of MMPC (data not shown).

### MCDC interfered with the cell cycle and AKT phosphorylation in MCF7 cells

**Figure 5:**
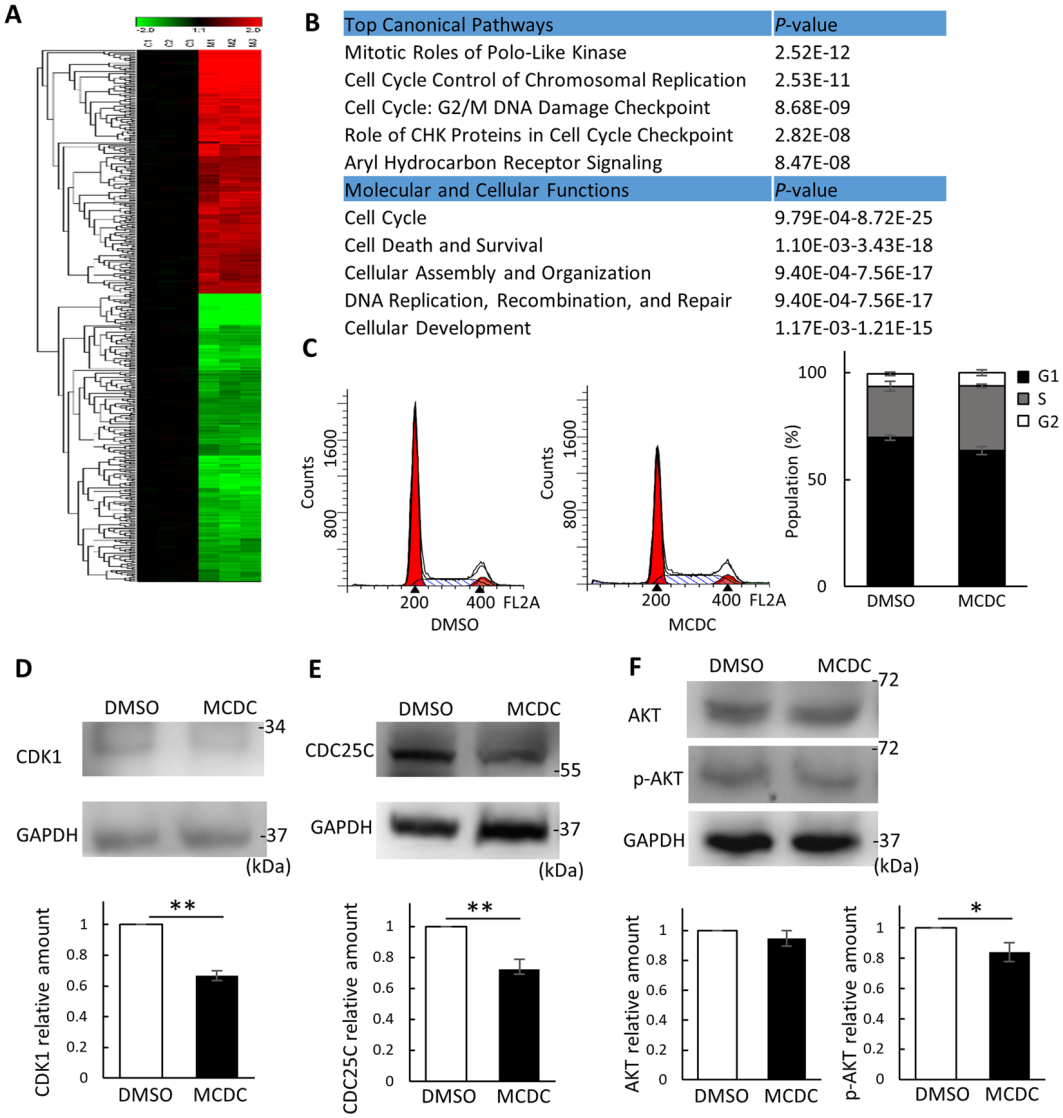
MCDC inhibits cell cycle-related protein expression and AKT phosphorylation in MCF7 cells. (**A**) Heatmap and hierarchical cluster analysis of differentially expressed genes in MCF7 cells treated with MCDC. MCF7 cells were treated with MCDC (5 μM) for 24 h. Total RNA was extracted, and genomic profiling was examined by Illumina Human HT-12 v4 BeadChips. Criteria for selecting MCDC-responsive genes: fold change > 2.5X and *P* < 0.05. (**B**) Results of Ingenuity Pathway Analysis of MCDC-responsive genes. (**C**) Cell cycle analysis on MCF7 cells after MCDC treatment. MCF7 cells were stained with propidium iodide and subjected to flow cytometry analysis after 24 h of treatment with 6 μM MCDC. The stacked bar chart summarizes three independent cell cycle experiments. (**D**–**F**) Immunoblots of S phase-related proteins. The bar charts incorporate data from three independent western blots. (**D**) CDK1, (**E**) CDC25C, and (**F**) MIF downstream protein AKT and phospho-Ser473 AKT, all at 48 h after treatment. All data are presented as means ± SDs of three independent experiments. *P < 0.05; **P < 0.01 by Student’s t-test.

Since MCDC was a newly synthesized compound, most of its functions, except its cytotoxic effects, remained unknown. Therefore, genome-wide profiling of MCF7 cells treated with MCDC (5 μM) was executed by using Illumina Human HT-12 v4 BeadChips. The dose of 5 μM was chosen because it was close to the IC50 value in MCF7 cells. By the selection criteria of *P* < 0.05 and fold changes of at least 2.5, 452 genes responding to MCDC treatment were identified. The expression profiles and clusters of differentially expressed genes are shown in Fig. 5A. Pathway analysis of these MCDC-responsive genes revealed that three of the five canonical pathways were cell cycle-related pathways and cell cycle was the first of all cellular functions (Fig. 5B). Based on these findings, we examined cell cycle phenotype by flow cytometry. The S phase was elevated by about 10% after treatment with 6 μM MCDC for 24 h, indicating S phase arrest in MCF7 cells (Fig. 5C). In addition, the S phase-related proteins CDK1 and CDC25C were examined in MCF7 cells after MCDC treatment. CDK1 expression levels decreased by ~35% after MCDC treatment (Fig. 5D) and CDC25C levels by ~30% (Fig. 5E).

Lastly, previous studies indicated that the AKT pathway is one of the downstream regulatory pathways of MIF^22–24^. AKT expression levels were similar between experimental groups and controls, but phosphorylation levels of AKT were inhibited by MCDC treatment by ~15% (Fig. 5F). These results suggest that MCDC decreased the cell viability of MCF7 breast cancer cells by decreasing the phosphorylation of AKT and inhibiting S phase of the cell cycle.

## Discussion

Identifying the direct target protein of a newly discovered or synthesized compound is often effort-intensive when using the typical methods of small molecule affinity chromatography^4^ and activity-based protein profiling^25^. These tools are time-consuming and limited in resolution, in part because the small molecular structures usually need to be modified, which results in changes in binding affinity with every new structure. In this study, we reported a rapid method to identify MIF as a possible direct binding protein for a new synthesized molecule, MCDC, by computational methods including structural and genomic screening. Firstly, the affinity binding of MCDC to MIF was shown by surface plasmon resonance technique. Secondly, the viability of U251 cells overexpressing MIF-N6Q/T7M mutants increased when cells were treated with different doses of MCDC. Thirdly, the protection against hydrogen-deuterium exchange assays showed that the fragment (1–79 amino acid) of MIF containing N6 was responsible for the binding with MCDC. Lastly, functional assays showed that MCDC inhibited the cell cycle by inhibiting the phosphorylation of AKT and the expression of S phase-related proteins.

Previously, we isolated and identified a panel of natural manzamine-related compounds, members of the β-carboline alkaloids, from marine origins, and some of them had cytotoxic effects against cancer cells^8^. In this study, chemical modification of these natural compounds was executed to simplify their structure and potentiate their cytotoxicity. Among the twelve synthesized manzamine derivatives, MCDC was chosen to demonstrate a time-saving workflow to identify the potential direct target proteins of a newly synthesized compound.

In preference to the slow processes of affinity chromatography^4^ and activity-based protein profiling^25^ to identify target proteins of small molecules, we used a bioinformatics approach to expedite this process by virtual docking. First, idTarget was used to search for potential targets of MCDC by screening all deposited proteins in PDB^20^. The potential target proteins were predicted by calculating the free energy of docking the input compound structure with all proteins in the PDB. This program provides the docking between one chemical and many proteins, but this method is limited to only those proteins that have been crystalized, and whose structures have been analyzed and deposited in the PDB. MIF (PDB No. 3l5 s) was the human protein with the lowest free energy when docked with MCDC.

Furthermore, to boost our confidence regarding the selection of target proteins of MCDC, another program, Discovery Studio, was used to predict the potential binding site of MCDC on MIF. Both Discovery Studio and idTarget predict docked structural complexes by computing difference in free energy between two different conformations. Other software, such as Connectivity Map (CMAP)^26^, uses different computational strategies to identify target proteins by comparing the genomic profiling of a tested compound to those of compounds with known targets in the database. We did not use CMAP in this study because the preliminary data showed that the function of compounds with high similarity to MCDC varied substantially, which made it difficult to design experiments to identify a direct target protein.

Our results showed that MCDC potentially bound at Asn6 of MIF via a hydrogen bond (Fig. 2). Yet, mutation of Asn6 to alanine did not decrease the binding affinity between MCDC and MIF-N6A (data not shown), but worsen the viability of U251 cells (Supplementary Fig. S3.). We speculated that the mutation from Asn (N) to Ala (A) probably increased the interspace and lead to better binding between MIF-N6A and MCDC. Therefore, we repeated the same experiments by constructing a new mutation to minimize the interspace by changing 6th and 7th amino acids to those with similar acidity and polarity but larger molecular size, i.e., N6Q and T7M. Mutation of Asn6 to Gln and Thr7 to Met resulted in increased viability of U251 cells upon treatment with MCDC (Fig. 3D), suggesting that the MIF-MCDC conjugate was toxic for U251 cells and showing that MIF was a valid target protein of MCDC. Although Discovery Studio can predict the docking site *in silico*, biological experiments are still needed to accurately locate the binding site. Therefore, HDX assays were used to examine the location of MIF’s interaction with MCDC at a per-peptide resolution (Fig. 4). We observed a relative large area of amino acids 1–79 of MIF had the protective effect against hydrogen-deuterium exchange assays. We explained that the phenomenon might be due to MIF itself a small protein, and most peptides in a small protein could be affected by binding to a small molecule in the H-D exchange rate.

Next, in order to understand the effect of MCDC on cellular function, genomic profiling of MCF7 cells treated with MCDC was performed by microarrays. The transcriptional levels of *MIF* did not significantly change upon MCDC treatment (data not shown), suggesting MCDC only affects the amount or conformation of MIF protein to initiate downstream signaling. Functional analyses of MCDC-responsive genes revealed that most of the differentially expressed genes were cell cycle-related (Fig. 5B). The decrease of cell cycle-related proteins was also observed by western blotting (Fig. 5D,E). These results suggest that the main effect of MCDC is inhibiting the cell cycle of tumor cells.

Examining the cytotoxic effects of MCDC in animal models will be a crucial step for future clinical applications. Yet, there are still some unresolved issues before *in vivo* experiments can be performed. First, the solubility of MCDC is too low in water because this compound is highly hydrophobic. In our studies, the organic solvent DMSO was used to dissolve MCDC. However, DMSO was not an ideal solvent for this system because it strongly interfered the H-D exchange. Also, DMSO is not suitable for oral and intravenous administration in humans. Therefore, in the HDX experiments, MMPC served as vehicle for MCDC, but for use with animal models or in clinical applications, the solubility of MCDC needs to increase. One possible solution is to add a hydroxyl group on a non-interacting site of MCDC.

Secondly, in addition to MIF, MCDC may have other target proteins. For example, matrix metalloproteinases MMP13 and MMP12 were also predicted as possible target proteins by idTarget (Table 3). MMP13 and MMP12 are involved in carcinomas and inflammatory conditions^27,28^. Also, the MIF downstream protein, AKT, was only partially blocked by MCDC, but MCDC had very potent cytotoxic effects against many cell lines (Supplementary Fig. S2 and Fig. 5F). Previous studies showed many MIF inhibitors had been developed and some of these inhibitors, such as ISO-1, had IC50 values similar to MCDC^29–32^. Therefore, we probably need to identify other possible target proteins, such as MMP13 and MMP12, and their pathways for MCDC in cell lines before performing *in vivo* experiments.

In summary, our work has highlighted a possible new MIF inhibitor, MCDC, by a rapid method of identifying potential target proteins for a newly synthesized compound. Based on the outcomes of this method, MCDC exerts cytotoxic effects against many cancer cell lines by inhibiting the downstream pathways of MIF.

## Materials and Methods

### Organic synthesis of 12 β-carboline derivatives

#### *N*-Alkylation

*9H*-carbazole (1.0 g, 5.98 mmol) was suspended in a mixture of toluene (8.0 mL) and 12 M aq. NaOH (8.0 mL). After stirring for 10 min, tetrabutylammonium iodide (220 mg, 0.60 mmol) was added to the reaction mixture. Alkyl bromide (2–3 equiv) was added and the reaction mixture was refluxed up to consumption of all starting material. The resulting crude product was carried into next step without purification (99% yield).

#### Vilsmeier-Haak reaction

*N*-alkyl carbazole (1.0 g, 5.50 mmol) was dissolved in anhydrous DMF (6.0 mL) under a nitrogen atmosphere. To that, phosphorus oxychloride (1.69 g, 11.04 mmol) was added. The reaction mixture was heated at 80 °C for 2–3 h to afford 9-alkyl-9H-carbazole-3-carbaldehyde (85% yield).

#### Pictet-Spengler cyclization

9-alkyl-9H-carbazole-3-carbaldehyde (1.0 g, 4.77 mmol) and tryptamine (0.92 g, 5.73 mmol) were suspended in glacial acetic acid (50 mL). The reaction mixture was heated to 80 °C for 2–3 h to afford tetrahydro-β-carboline as a brown solid (1–6, 80% yield).

#### Oxidation

*N*-Substituted tetrahydro-β-carboline (1.0 g, 2.84 mmol) was dissolved in anhydrous THF under a nitrogen atmosphere. To that, KMnO4 (0.67 g, 4.27 mmol) was added and then stirred at 0 °C for 2 h to afford dihydro-β-carboline (imine) as a yellow solid (7–12, 78% yield).

### Cell culture

Cancerous lung adenocarcinoma cell lines, including A549 and H1299 cells, were cultured in RPMI medium 1640 (GIBCO, Carlsbad, CA, USA) with 1% streptomycin/puromycin (Biological Industries, Cromwell, CT, USA) and 10% fetal bovine serum (Biological Industries, Beit-Haemek, Israel). The cultured plates were maintained at 37 °C in a humidified atmosphere with 5% CO2. Liver hepatocellular cell line HepG2 and breast adenocarcinoma cell line MCF7 were cultured in DMEM medium (GIBCO) in the same conditions. Glioblastoma-astrocytoma U251 cells were cultured in MEM medium (GIBCO) in the same conditions.

### Calcein AM viability assay

A549, H1299, HepG2, and MCF7 cells were seeded at a cell density of 3,000 cells per well in black opaque 96-well microplate. Cells were treated with 1–100 μM of the 12 new synthesized compounds 24 h after seeding. The Calcein AM cell viability assay, including concentration and reading wavelength, was conducted on each cell type according to the manufacturer’s instructions (Biotium, Fremont, CA, USA). The fluorescence intensity (Ex/Em: 494/517 nm) was measured 48 h after addition of the small molecules using a SpectraMax Paradigm Multi-Mode Microplate Reader (Molecular Devices, Sunnyvale, CA, USA).

### MTT proliferation assay

Cells were seeded at a density of 3,000 cells per well in 96-well microplate and treated with 2–8 μM 1-(9′-methyl-3′-carbazole)-3, 4-dihydro-β-carboline (MCDC) 24 h after seeding. The testing concentration of 3-(4, 5-dimethylthiazol-2-yl)-2, 5-diphenyltetrazolium bromide (MTT; BioTek, Winooski, VT, USA) was 0.456 mg/mL. Cells were kept at this concentration of MTT at 37 °C, 5% CO2 for 1.5 h, then dissolved in 100 μL DMSO per well. The absorbance values were measured 48 h after MCDC treatment using a microtiter plate reader (BioTek) at 570 nm.

### *In silico* screening for potential target proteins

The idTarget website (http://idtarget.rcas.sinica.edu.tw/) was used to screen all potential proteins in the Protein Data Bank (PDB) for potential interaction with MCDC. We drew MCDC’s molecular structure online using idTarget’s built-in program. Other docking and subsequent scoring parameters were kept at the default settings.

### Binding site analysis

The docking tool Discovery Studio 3.0 (Accelrys/Biovia, San Diego, CA, USA) was used to calculate and edit the docking conformation between macrophage migration inhibitory factor (MIF) and MCDC. The standard default settings were used in the calculations, but the minimum site size was set to 500. Conformations of the docked complex were visualized and color-coded by PyMOL 2.0 (pymol.org).

### Surface plasmon resonance analysis

The real-time binding interaction of MIF with MCDC was measured by surface plasmon resonance using a Biacore T200 sensor (Biacore GE, Uppsala, Sweden). Series S sensor chip CM7 (GE, Buckinghamshire, UK) was served as the detector chip. The recombinant human MIF protein (PeproTech, NJ, USA) was immobilized on the detector chip according to the manufacturer’s instructions by an amine coupling kit (GE, Buckinghamshire, UK) at the pH 5.5. The derived sensor chips were washed by the Biacore running buffer, PBS-P (GE, Uppsala, Sweden). Binding was measured at 25 °C and flow rate 30 μl/min. One minute of sensor chip regeneration was performed with PBS-P. The whole process was repeated three times. Sensorgram response data were analyzed by the Biacore evaluation kinetics package to calculate the equilibrium dissociation constants.

### Hydrogen-deuterium exchange/mass spectroscopic analysis

The hydrogen-deuterium exchange (HDX) of MIF recombinant human protein (GenScript, NJ, USA) was measured in the presence and absence of MCDC. The stock of MCDC was dissolved in 10% 1-myristoyl-sn-glycero-3-phosphocholine (MMPC), or in 15% dimethyl sulfoxide (DMSO) for a control. The MIF protein (15 pmol) and MIF-MCDC complex (15 pmol MIF per 150 or 1,500 pmol MCDC) samples were diluted in exchange buffer (99.9% D2O in phosphate-buffered saline [PBS], pH 7.4) at a 1:10 ratio to initiate HDX at room temperature. At 5 time points (15, 45, 135, 405, and 1,215 s), an aliquot (1.5 pmol of target protein) was aspirated and mixed with pre-chilled quenching buffer (final concentration of 1.5 M guanidine hydrochloride, 150 mM tris(2- carboxyethyl)phosphine, 0.8% formic acid, and 0.013% MMPC or 0.15% DMSO). The mixture was immediately loaded onto homemade pepsin column for digestion of MIF. The digested peptide mixtures were then loaded onto a reverse-phase HPLC column (Zorbax 300SB-C18, 0.35 mm; Agilent Technologies, Wilmington, DE, USA). The desalted peptides were separated on a homemade column (HydroRP 2.5 μm, 75 μm I.D. 5.5 cm) using a linear gradient (8–95%) of HPLC buffer (99.9% acetonitrile/0.1% formic acid/0.025% trifluoroacetic acid) for 10 min with a flow rate of 0.5 μL/min. This chromatography apparatus was coupled with a 2D linear ion trap mass spectrometer (Orbitrap Classic; Thermo Fisher, San Jose, CA, USA) operated by Xcalibur 2.0.5 software (Thermo Fisher). The full-scan MS was performed in the Orbitrap over a range of 350 to 1,600 Da and a resolution of 60,000 at m/z 400. Internal calibration was performed using the ion signal of [Si(CH3)2O]6H+ at m/z 536.165365 as lock mass. The electrospray voltage was set at 2.2 kV and the temperature of the capillary was set at 200 °C. MS and MS/MS automatic gain controls were set at 1,000 ms (full scan) and 120 ms (MS/MS), or 2 × 10^6^ ions (full scan) and 3 × 10^3^ ions (MS/MS) for maximum accumulation time or ions, respectively.

### Peptide identification and hydrogen-deuterium exchange data analysis

The peptide identification was carried out using Proteome Discoverer software (version 1.4, Thermo Fisher). The MS/MS spectra were searched against the single protein database using the SEQUEST search engine. For peptide identification, a 10 ppm mass tolerance was permitted for intact peptide masses and 0.5 Da for collision-induced dissociation into fragment ions. Peptide-spectrum matches were then filtered based on confidence level and search engine rankings of 1 for peptide identification to ensure an overall false discovery rate below 0.01. For HDX analysis, the peptide identification template was made from the LC-MS/MS results of the unexchanged target protein. The template was then preloaded in the ExMS module of MATLAB. The MS spectrum after HDX was loaded and analyzed to calculate the number of incorporated deuteriums for each peptide, which was then presented as the average deuterium incorporation of two independent experiments.

### Endogenous *MIF* expression in multiple cell lines

Previously, our research team developed a gene expression analysis system, Cellexpress This system is available online (http://cellexpress.cgm.ntu.edu.tw/). We searched MIF expression levels in cell lines in the Sanger Cell Line Project dataset, normalized against β-actin expression.

### *MIF* overexpression system and site mutation

Full-length *MIF* cDNA (348 bp) tagged with a Flag epitope was inserted into a pcDNA3.1 + C-DYK expression vector. All sequences were verified by Sanger sequencing (First Core Laboratory, College of Medicine, National Taiwan University). This plasmid was transiently transfected into U251 cells at the same time as seeding. The transfection amount was in accordance with the following conditions: 250 ng plasmid DNA, 12.5 µL jetPRIME buffer, and 0.25 µL jetPRIME solution (Polyplus, NY, USA) for 1 mL medium with cells.

The seventh and eighth amino acid of MIF was changed from asparagine and threonine to glutamine and methionine respectively using the following primers: Forward, 5′- TGCCGATGTTCATCGTACAAATGAACGTGCCCCG-3′; Reverse, 5′-CGGGGCACGTTCATTTGTACGATGAACATCGGCA-3′. Site-directed mutagenesis was carried out with restriction enzyme *Dpn* I (NEB, MA, USA) and High-Fidelity PCR Master Mix (Thermo Fisher) according to the manufacturers’ instructions.

### RNA extraction and quantitative RT-PCR

Total RNA was extracted by TRIpure reagent (Roche Diagnostics, Branchburg, NJ, USA) according to the manufacturer’s protocol. One μg of total RNA was reverse-transcribed to cDNA using a High-Capacity cDNA Reverse Transcription kit (Applied Biosystems, Carlsbad, CA). Ten percent of each cDNA was used as the template. mRNA levels were quantified by qRT-PCR using *MIF*-specific primers (Forward: 5′-CTCTGCAGCCTGCACAGCAT-3′ and Reverse: 5′-GGTGGAGTTGTTCCAGCCCAC-3′). The SYBR Green intensity was measured by StepOnePlus Real-Time PCR Systems (Thermo Fisher) and OmicsGreen qPCR 5X Master Mix (OmicsBio, Taipei, Taiwan) according to the manufacturer’s instructions.

### Human genome microarray analysis and Ingenuity Pathway Analysis

The total RNA was primed with T7 Oligo(dT) primer and amplified using a Illumina TotalPre RNA Amplification kit (Ambion Inc., Austin, TX, USA) to synthesize the cDNA containing a T7 promoter sequence. Thereafter, *in vitro* transcription was conducted using the double-stranded cDNA as a template and T7 RNA polymerase to synthesize multiple copies of biotinylated cRNA. After amplification, the cRNA was mixed with an equal volume of hybridization buffer and hybridized to Illumina Human HT-12 v4 BeadChips (Illumina) at 58 °C for 16 h. After hybridization, the BeadChip was washed and stained with streptavidin-Cy3 dye. The intensity of the bead’s fluorescence was detected by the Illumina BeadArray Reader, and the results were analyzed using BeadStudio v3.1 software. After scanning, the intensity data of Illumina Human HT-12 v4 BeadChips were analyzed using the commercial software Partek (Partek, St. Charles, MO, USA) for mRNA analysis. Background-adjusted signals were normalized by a quantile normalization algorithm, which normalized the probe intensities based on the intensity distribution among all slides. Furthermore, Ingenuity Pathway Analysis (Ingenuity Systems Inc., Redwood City, CA, USA) was applied to comprehend gene-gene interaction networks, biological functions, and pathways of the differentially expressed genes.

### Cell cycle analysis

Cells were harvested by trypsinization, washed with PBS (GIBCO), and fixed with cold 100% ethanol at −20 °C overnight. After removing the ethanol, cell pellets were resuspended in PBS containing 20 µg/mL propidium iodide (Life Technologies, Eugene, Oregon, USA), 0.1% Triton-X-100 (Sigma, Saint Louis, MO, USA) and 100 µg/mL RNase A (Sigma) for 15 min. The suspension was passed through a nylon mesh filter and analyzed using Beckman Coulter FC500 (Beckman, Brea, CA, USA) and ModFit LT analysis software.

### Protein extraction and western blotting

Cells were lysed in RIPA lysis buffer (Sigma) and protein concentration was determined using protein assay reagent (Bio-Rad Laboratories, Hercules, CA, USA). Protein lysate was separated by 10% sodium dodecyl sulfate-polyacrylamide gel electrophoresis (SDS-PAGE) and transferred to a polyvinylidene difluoride (PVDF) membrane (Bio-Rad Laboratories). Membranes were blocked in T-Pro Fast Blocking Buffer (T-Pro Biotechnology, Taiwan) for 10 min and hybridized to a primary antibody.

After immunoblotting, the membranes were washed with tris-buffered saline (OmicsBio, Taipei, Taiwan) containing 0.1% Tween 20 and reacted with horseradish peroxidase-conjugated goat anti-rabbit IgG (GeneTex, Irvine, CA, USA). The protein bands were visualized using an enhanced chemiluminescence system (Millipore, Billerica, MA, USA). Western blot images were analyzed by Gel-Pro Analyzer software (Meyer Instruments, Houston, TX, USA). The primary antibodies we used were as follows: rabbit anti-human CDK1, CDC25C, AKT, and AKT (phospho-Ser473) polyclonal antibodies (GeneTex, Irvine, CA, USA).

## Supplementary information

Supplementary Information
