# Supplementary material for: Macrophage Migration Inhibitory Factor Acts as the Potential Target of a Newly Synthesized Compound, 1-(9′-methyl-3′-carbazole)-3, 4-dihydro-β-carboline
Source: Sci Rep. 2019 Feb 14;9:2147. doi: 10.1038/s41598-019-38590-y (PMC6375994; doi:10.1038/s41598-019-38590-y)
Supplement: Supplementary file 1 — Supplementary Information [file 41598_2019_38590_MOESM1_ESM.pdf]

Macrophage Migration Inhibitory Factor Acts as the Potential Target of a Newly Synthesized Compound, 1-(9'-methyl-3'-carbazole)-3,4-dihydro-β-carboline

Pin-Hao Ko[1†], Ya-Ching Shen[2†], Kaliyappan Murugan[2], Chiung-Wei Huang[3], Govindan Sivakumar[2], Pinki Pal[2], Chia-Ching Liao[2], Kai-Shin Luo[4], Eric Y. Chuang[5,6], Mong-Hsun Tsai[6,7], Liang-Chuan Lai[1,6]*

[1]Graduate Institute of Physiology, National Taiwan University, Taipei, Taiwan; [2]School of Pharmacy, College of Medicine, National Taiwan University, Taipei, Taiwan; [3]Department of Physiology, College of Medicine, Kaohsiung Medical University, Kaohsiung, Taiwan; [4]Department of Ophthalmology, Taipei Tzu Chi Hospital, Buddhist Tzu Chi Medical Foundation, Taipei, Taiwan; [5]Graduate Institute of Biomedical Electronics and Bioinformatics, National Taiwan University, Taipei, Taiwan; [6]Bioinformatics and Biostatistics Core, Center of Genomic Medicine, National Taiwan University, Taipei, Taiwan; [7]Institute of Biotechnology, National Taiwan University, Taipei, Taiwan

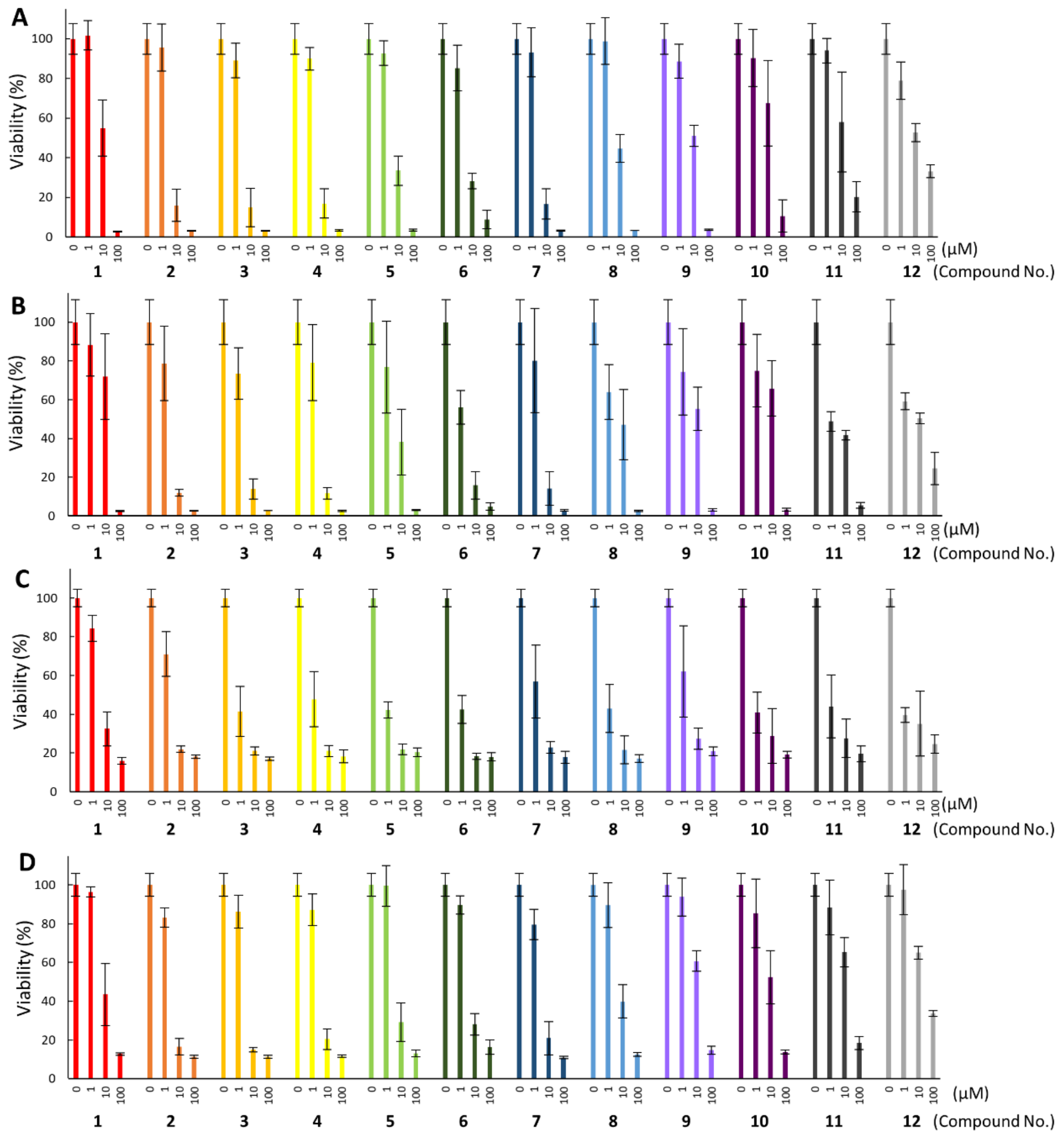

Supplementary Figure S1. Cytotoxicity of 12 β-carboline derivatives in four cancer cell lines. Viability of A549 (A), H1299 (B), HepG2 (C) and MCF7 (D) cells was measured by Calcein AM assays 48 h after treatment at the wavelength of (Ex/Em: 494/517 nm). All experiments were repeated at least three times. All data are presented as means ± SDs of three independent experiments.

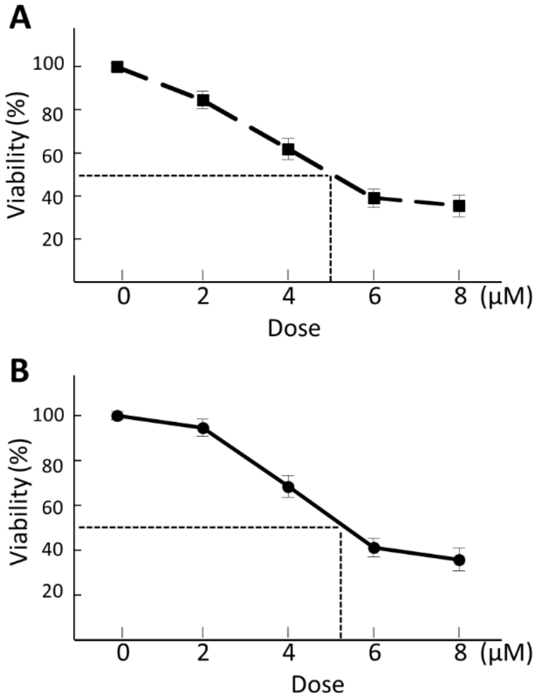

Supplementary Figure S2. IC50 of 1-(9'-methyl-3'-carbazole)-3,4-dihydro-β-carboline (MCDC) in A549 and MCF7 cells. Cytotoxic effects of MCDC on A549 (A) and MCF7 (B) cells measured by MTT assays at 48 h of treatment. IC_{50} represents the concentration of MCDC at which 50% of cells are viable. All experiments were repeated at least three times. All data are presented as means ± SDs of three independent experiments.

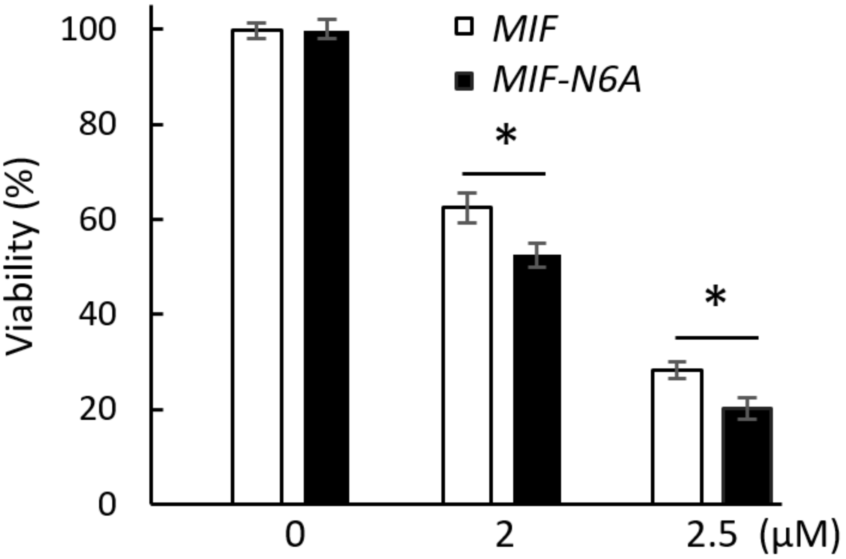

Supplementary Figure S3. Effects of MIF-N6A mutant on viability of U251 cells treated with MCDC. The viability of U251 cells overexpressing MIF or MIF-N6A under different doses of MCDC was measured by MTT assays 48 h after treatment. All data are presented as means ± SEM. *, *P* <0.05; **, *P* <0.01 by *Student's t-test.*

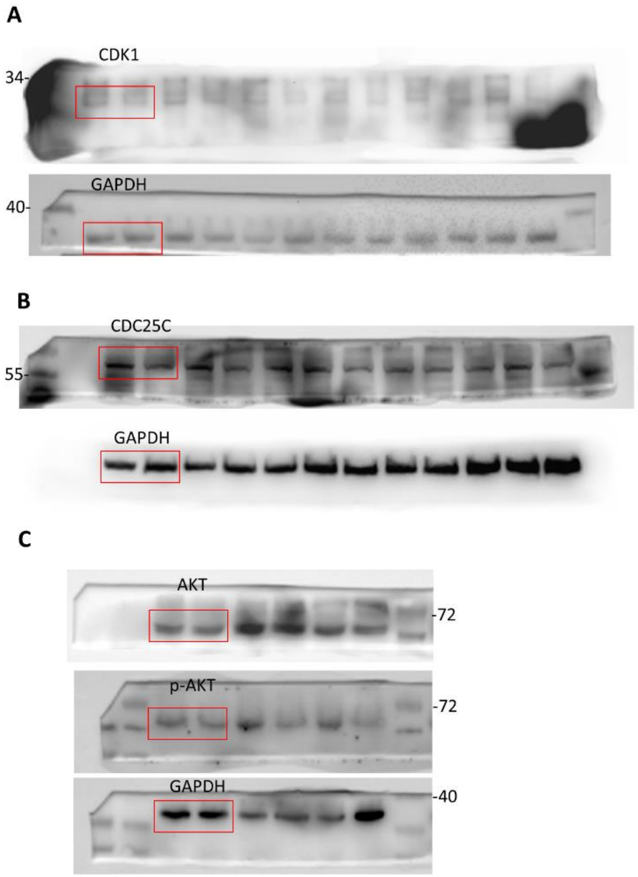

Supplementary Figure S4. Uncropped images of blots presented in (A) main Figure 5D, (B) main Figure 5E and (C) main Figure 5F. Total protein were harvest from MCF7 cells after MCDC treatment for 48 h.
